# A Rare Case of Hansen’s Disease Complicated by G6PD Deficiency

**DOI:** 10.7759/cureus.42816

**Published:** 2023-08-01

**Authors:** Payton Yerke Hansen, Elisha Myers, Karan Rajalingam, Mary Labanowski

**Affiliations:** 1 Surgery, Florida Atlantic University Charles E. Schmidt College of Medicine, Boca Raton, USA; 2 Internal Medicine, Florida Atlantic University Charles E. Schmidt College of Medicine, Boca Raton, USA

**Keywords:** coinfection, glucose-6-phosphate-dehydrogenase deficiency (g6pd), tuberculosis, leprosy, hansen’s disease

## Abstract

We present the case of a 37-year-old Haitian male who presented with a seven-month history of skin lesions on his face and extremities, weight loss, intermittent chills, difficulty in breathing, and bilateral paresthesias in his feet. The lesions were most prominent on the pinnae of the ears. Biopsy of the lesions revealed large, rounded granulomatous infiltrates and histiocytes. Acid fast (Ziehl-Neelsen technique) and Kinyoun stains were positive for numerous acid-fast mycobacteria within the histiocytes. A polymerase chain reaction (PCR) was positive for *Mycobacterium leprae, *which confirmed a diagnosis of lepromatous leprosy. Further analysis revealed positive purified protein derivatives (PPD) and QuantiFERON-TB™ test (QIAGEN, Hilden, Germany) with negative chest x-ray and sputum cultures. Labs also revealed vitamin D and G6PD (glucose-6-phosphate-dehydrogenasedeficiency. The patient was started on a combined therapy regimen of rifampin, moxifloxacin, and minocycline. In addition, he was started on vitamin D supplementation. After undergoing treatment for one year, there was notable regression of the patient’s cutaneous lesions. Treatment is planned to continue for a total of 24 months. This case exemplifies the successful treatment of Hansen’s disease in a patient with a G6PD deficiency. The patient's G6PD deficiency required avoidance of dapsone, which is typically used in the treatment of Hansen’s disease. Furthermore, the patient’s positive PPD and QuantiFERON-TB tests led to a delay in the treatment in order to rule out active tuberculosis. Left untreated, Hansen’s disease has a high morbidity risk. Treatment regimens require careful consideration of coexisting comorbidities.

## Introduction

Hansen’s disease, also known as leprosy, is an infectious disease caused by *Mycobacterium leprae* that primarily affects the skin and peripheral nerves [1]. It is highly contagious and transmitted through respiratory droplets from an infected individual. Furthermore, individuals with multibacillary leprosy have higher transmission rates [2]. While better global control of the disease has been accomplished through multidrug therapy and vaccination, Hansen’s disease remains endemic to over 140 countries with more than 200,000 new cases detected in 2019 [3,4]. While non-endemic in the United States, approximately 150 to 250 new cases are still diagnosed annually [5]. The majority of cases of Hansen’s disease in the United States are diagnosed in immigrants; however, locally acquired infections also occur [5-7]. Due to the rarity of the disease in United States residents, local infections are more likely to have a delay in diagnosis and treatment compared to infections in immigrants [5].

Additionally, patients suffering from Hansen’s disease often have long-term complications such as permanent neurologic sequelae and disfigurement [5]. Furthermore, 60% of patients who contract Hansen’s disease have peripheral nerve damage at the time of diagnosis, which leads to increased morbidity rates [4]. Therefore, the efficient diagnosis and treatment of leprosy is crucial. Comorbidities, such as glucose 6-phosphate dehydrogenase (G6PD) deficiency, must be considered in formulating a treatment regimen to prevent adverse effects and avoid antibiotic resistance.

Dapsone is commonly used in the treatment of Hansen’s disease. While relatively uncommon, dapsone releases N‐hydroxy metabolites that can cause hemolytic anemia. Concomitant G6PD significantly increases the risk of severe intravascular hemolysis. This can be associated with high morbidity rates [8,9]. Furthermore, the rates of G6PD deficiency range from 2.3% to 27% in areas that are endemic to Hansen’s disease. While the prevalence is unknown, there is a risk that these conditions can occur simultaneously [10]. We present a case of a 37-year-old Haitian male with Hansen’s disease whose treatment was complicated by G6PD deficiency.

## Case presentation

A 37-year-old Haitian male presented to a local free clinic with a seven-month history of intermittent fever and chills, dyspnea, and skin lesions on his extremities and face, most prominent on the pinnae of the ears. The skin lesions were sharply demarcated hypopigmented nodules with a scaly center and erythematous borders. The nodules were associated with hypoesthesia, but the patient denied pain or pruritus. He first noticed the painless nodules on his right hand while in an immigration camp in California. The number of nodules increased and progressed to his face and right leg. He also noted complaints of bilateral paresthesia in his feet, swelling and weakness in his hands, occasional blood in his sputum, palpitations, and weight loss of an unknown amount. He denied visual complaints, vomiting, nausea, or diarrhea. The patient's past medical history is unremarkable. He was born in Haiti but lived in the Dominican Republic, Brazil, and the United States. At the time of presentation, he was living alone and working as a landscaper in South Florida. Previous occupations included working at a market and as a construction worker. He denied any smoking or drug use but endorsed occasional alcohol use. The patient reported having more than 10 sexual partners in the last five years. His HIV and rapid plasma reagin (RPR) were negative three months ago. 

**Figure 1:**
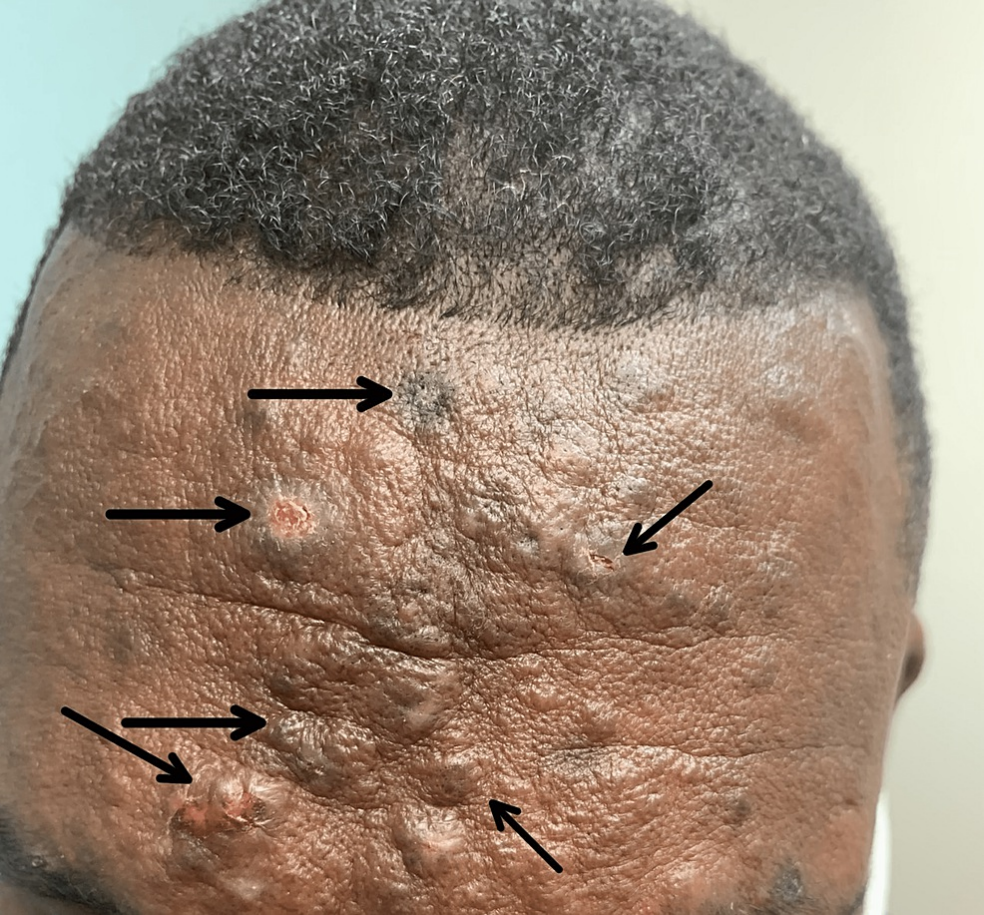
This patient presented with dark and erythematous raised lesions over his face, hands, arms, and legs. The image depicts the lesions on his forehead.

At the clinic, the patient appeared comfortable and in no distress. His vital signs were all within normal limits. His physical exam revealed extensive dark and erythematous raised lesions ranging in size from 0.5 to 3 cm over his face, hands, arms, and legs (Figure 1). The lesions were without exudate and were only minimally tender to palpation. His oropharynx was without lesions, but areas of hyperpigmentation were noted. Mild inflammation of the nasal mucosa was also observed. Cardiac, pulmonary, and abdominal exams were normal. The patient’s hands were noted to have swelling in all digits with a diminished grip bilaterally. No edema was observed in his lower extremities. His bicep and tricep reflexes were diminished on the left side. He also had diminished light touch on the plantar surface of his feet. Vibration sense was intact in both hands and feet. He also had palpable thickening of the common peroneal nerve.

Skin biopsy of lesions revealed large rounded granulomatous infiltrates composed of plump epithelioid histiocytes. A periodic acid-Schiff (PAS) stain was negative for fungal organisms. Acid-fast (Ziehl-Neelsen technique) and Kinyoun stains were positive for numerous acid-fast mycobacteria within the histiocytes. The bacterial index (BI) was 2+ and the morphological index (MI) was 40, indicating multibacillary leprosy.

A polymerase chain reaction (PCR) was positive for *Mycobacterium leprae* indicating lepromatous leprosy, which was subsequently confirmed by the National Hansen's Disease Program, a national organization that monitors leprosy in the United States. Per the recommendation of the National Hansen’s Disease Program, additional laboratory tests were completed that revealed a Vitamin D deficiency and G6PD deficiency. Further workup also revealed positive purified protein derivatives (PPD) and QuantiFERON-TB™ test (QIAGEN, Hilden, Germany). A chest x-ray did not show any changes characteristic of latent TB. This was further confirmed with negative sputum cultures. 

As per the National Hansen's Disease Program, the patient was started on a monthly combined therapy regimen of rifampin 600 mg, moxifloxacin 400 mg, and minocycline 100 mg, Vitamin D 50,000 IU once a week for at least 12 weeks, methotrexate 7.5 mg twice weekly, and folic acid 1 mg daily [11-15]. 

**Figure 2:**
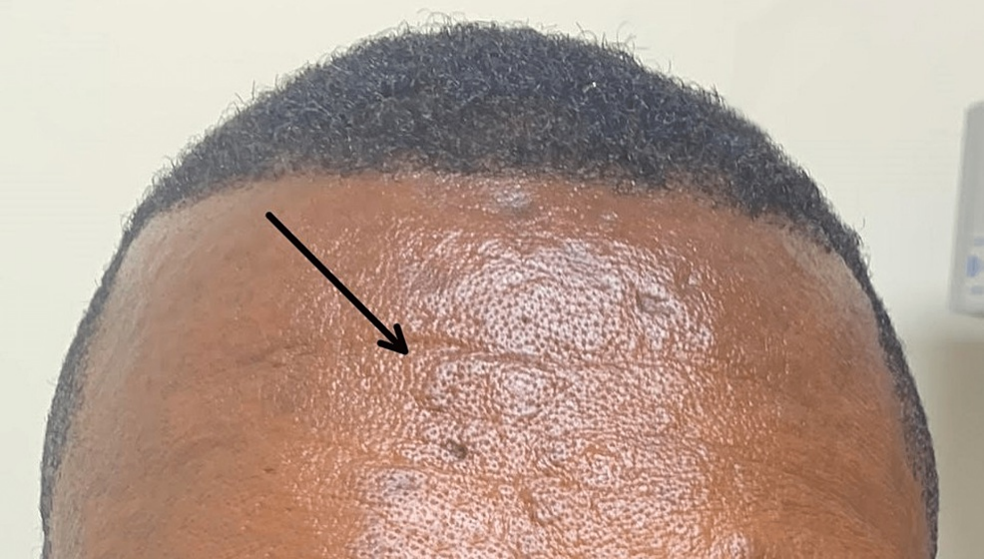
Notable regression of the lesions on his forehead after undergoing treatment for one year.

The patient responded well to treatment, with remarkable skin and joint improvements, which was continued over his first year of treatment (Figure 2). He experienced anorexia and weight loss, so methotrexate was discontinued. He was able to gain weight back after cessation of methotrexate and had no recurrent joint symptoms. Treatment is to be continued for a total of 24 months if tolerated. He will be further analyzed for a possible tuberculosis infection following this course.

## Discussion

This case illustrates the challenges in treating Hansen’s disease that is complicated by a an underlying G6PD deficiency. The World Health Organization (WHO) recommends a multidrug regimen for the treatment of Hansen’s disease. Patients with paucibacillary leprosy should receive rifampin 600 mg monthly and dapsone 100 mg daily for six months. In contrast, patients with multibacillary leprosy receive the same dosages of rifampin and dapsone for a 24-month duration. Additional dosages of clofazimine are also used in this patient population [16,17]. However, special considerations should be taken when choosing the appropriate therapy because coexisting conditions, such as G6PD, can lead to further complications and high mortality rates.

G6PD deficiency is an X-linked disorder resulting in an absent or deficient G6PD enzyme [18]. The G6PD enzyme plays a critical role in the conversion of nicotinamide adenine dinucleotide phosphate (NADP) to nicotinamide adenine dinucleotide phosphate hydrogen (NADPH) within the pentose phosphate pathway. This conversion is crucial in the production of glutathione, which is an antioxidant that protects erythrocytes from hemolysis due to oxidative stress [18,19]. In G6PD-deficient patients, hemolysis can occur due to reactive oxygen species and free radicals from environmental stressors such as medications, infections, and certain foods. The WHO classifies variants of G6PD deficiency into classes I-V according to the degree of enzyme deficiency and hemolysis severity. Class I is the most severe while classes IV and V are considered clinically silent [18-21]. Of the patients who have G6PD deficiency, 1.2% have Class I, 5.6% have Class II, 11.0% have Class III, 76.6% have Class IV, and 5.6% have Class V [22]. Due to the risk of hemolysis, patients are often tested for G6PD deficiency before starting contraindicated medications. In our patient, dapsone was contraindicated based on his G6PD status. Therefore, the patient was treated with a multidrug regimen consisting of rifampin, moxifloxacin, minocycline, and methotrexate for Hansen’s disease.

The multidrug regimen used in this case was given monthly to prevent side effects and improve compliance. Furthermore, the supplementation of vitamin D was used to prevent deficiencies, which is a known factor in the immune response [23]. While the traditional approach requires daily delivery of these, the National Hanson's Disease Program has had great success with a monthly regimen [11,24]. Franco-Paredes et al. utilized this monthly regimen for 12-24 months with satisfactory clinical outcomes and 100% patient compliance. Furthermore, they noted minimal side effects compared to the traditional daily regimen. In addition, patients with Hansen’s disease can have systemic inflammatory complications known as leprosy reactions. Typically, leprosy reactions are treated with prolonged corticosteroid therapy, which can cause severe side effects. Methotrexate can be used as a corticosteroid-sparing approach and has shown comparable results [24].

In addition to Hansen’s disease, our patient had positive PPD and QuantiFERON-TB tests. In cases of Hansen’s disease, PPD and QuantiFERON TB are not reliable tests for tuberculosis because both the early secreted antigenic target 6 kDa (ESAT6) and culture filtrate protein 10 (CFP10) antigens of *Myocobacterium leprae* are 100% cross-reactive with these tests [25]. Therefore, in a patient with Hansen’s disease and intact cell-mediated immunity, positive PPD and QuantiFERON tests may be due to *Myocobacterium leprae* ESAT6 or CFP10 rather than the *Myocobacterium tuberculosis* epitopes [25,26]. Therefore, further workup in this patient is warranted following the treatment for Hansen’s disease.

The simultaneous infection of TB and Hansen’s disease is rare, and occurrences range from 2.5% to 13.5% in endemic areas [27]. Coinfections warrant a specific treatment regimen to prevent future complications and disease transmission. The relationship between TB and Hansen’s disease remains unclear. Both diseases involve mycobacteria that cause chronic granulomatous diseases; however, Hansen’s disease primarily affects the peripheral nervous system and skin. In contrast, TB infections predominantly affect the lungs and disseminate to other organs [28]. Previously, researchers proposed that Hansen’s disease and TB were antagonistic and provided cross-immunity to protect against dual infections [28-30]. This proposal is supported by the bacille Calmette-Guerin (BCG) vaccine providing protection against both TB and Hansen’s disease [31,32]. The cross-immunity hypothesis is countered by cases of TB and Hansen’s disease coinfections. Other hypotheses state that Hansen’s disease suppresses the immune response and therefore increases the susceptibility to TB, which can lead to increased mortality rates. These higher mortality rates were confirmed by a study conducted by Rajagopala et al., which found a 37.2% mortality rate associated with TB and Hansen’s disease coinfections [27]. Furthermore, they found that TB was most associated with borderline lepromatous leprosy and lepromatous leprosy [27,28]. Risk factors associated with coinfections include diabetes mellitus, malnutrition, chronic kidney disease, and corticosteroid use [3,27]. In contrast, our patient reported no premorbid illnesses nor was he undergoing immunosuppressant treatments. Furthermore, he had a negative chest x-ray.

By recognizing the potential for multiple underlying conditions and being proactive with screening, clinicians can vastly improve patient outcomes while reducing healthcare costs. This is exemplified in our case. The recommended treatment for Hansen’s disease could have caused hemolytic anemia in this patient due to his G6PD deficiency. Finally, prompt treatment reduced the risk of disease transmission.

## Conclusions

Early detection and treatment of Hansen’s disease is crucial in reducing complications and further transmission. Furthermore, the management of leprosy is challenging even for experienced clinicians because comprehensive treatment requires multispecialty care coordination to treat the various sequelae of leprosy. Additionally, the National Hansen’s Disease Program provided essential guidance in facilitating treatment. The need for coordinated care is further amplified in patients with other underlying conditions that can complicate their disease course. This case exemplifies the difficulty in treating Hansen’s disease in a patient with G6PD deficiency, which required avoidance of certain medications typically used to treat Hansen’s disease. Furthermore, the cross-reactivity between Hansen’s disease and tuberculosis can cause false positive PPD and QuantiFERON-TB tests. Therefore, these tests are unreliable in patients with leprosy. Left untreated, Hansen’s disease can result in significant medical morbidities, and treatment regimens require careful consideration of coexisting comorbidities. 
